# The potential role of *P.gingivalis* in gastrointestinal cancer: a mini review

**DOI:** 10.1186/s13027-019-0239-4

**Published:** 2019-09-10

**Authors:** Xiao-bo Liu, Zi-ye Gao, Chuan-tao Sun, Hui Wen, Bo Gao, Sheng-bao Li, Qiang Tong

**Affiliations:** 1 Department of Gastroenterology, Taihe Hospital, Hubei University of Medicine, 32 south renmin road, Shiyan, Hubei 442000 People’s Republic of China; 2 Department of Oncology, Taihe Hospital, Hubei University of Medicine, Shiyan, 442000 Hubei China; 3 Department of Laboratory Medicine, Taihe Hospital, Hubei University of Medicine, Shiyan, 442000 Hubei China

**Keywords:** *Porphyromonasgingivalis*, Periodontitis, Gastrointestinal cancer;esophageal cancer, Pancreatic cancer

## Abstract

Bacterial infection may be involved in the entire process of tissue carcinogenesis by directly or indirectly affecting the occurrence and development of tumors. *Porphyromonas gingivalis (P.gingivalis)* is an important pathogen causing periodontitis. Periodontitis may promote the occurrence of various tumors. Gastrointestinal tumors are common malignant tumors with high morbidity, high mortality, and low early diagnosis rate. With the rapid development of molecularbiotechnology, the role of *P.gingivalis* in digestive tract tumors has been increasingly explored. This article reviews the correlation between *P.gingivalis* and gastrointestinal cancer and the pathogenesis of the latter. The relationship among *P.gingivalis*, periodontal disease, and digestive tract tumors must be clarifiedthrough a multi-center, prospective, large-scale study.

## Introduction

Pathogen infection is one of the main causes of chronic inflammation, and the most common types are bacterial and viral infections [1]. With the development of clinical medicine, a fundamental has shift occurred in the link between bacteria and cancer [2]. Tumorigenesis may be associated with inflammation. Bacterial infection may participate in the entire process of carcinogenesis by directly or indirectly affecting the occurrence and development of the disease. [1] Chronic infection may be involved in 17~28% of the morbidity and death of patients with cancer in countries such as China and South Korea [3].

The human flora plays an important role in normal physiological activities [4], and carcinogenesis [5]. More than 700 kinds of bacteria exist in the normal oral cavity, and these include at least 11 mycobacteria and 70 genera [6], among which *red complex* is an important pathogenic bacterium for periodontal disease [7]. *Red complex* consists of *P.gingivalis*, *Treponema denticola (T. denticola)* and *Tannerellaforsythia* [7]. All three are Gram-negative anaerobic bacteria, which can express virulence factors to interfere in the defense system,and invade and destroy periodontal tissue and host immune function [7].

The morbidity rate of periodontitis is approximately 10.0~17.6%, and it is often accompanied with increasing levels of inflammatory factors [8]. The definition “chronic periodontitis” has been replaced in the new periodontal diseases classification [9], the forms of the disease previously recognized as “chronic” or “aggressive”, now grouped under a single category, “periodontitis”. In this study we have made changes according to the latest standards. Pathogenic bacteria can spread to the blood and even to the brain [10], and they are associated with various of systemic diseases and cancers [11, 12]. Periodontitis can cause tooth loss, and meta-analyses [13] suggest that tooth loss and alimentary canal cancer are remarkbaly correlated and exhibit a dose–effect relationship, that is, the overall risk of cancer increases by 9% for every 10 teeth lost, and esophageal cancer (+ 14%), gastric cancer (+ 9%), head and neck cancer (+ 31%), colorectal cancer (+ 4%) and pancreatic cancer (+ 7%) [13].

*P.gingivalis* is an important pathogenic bacterium that mediates the local inflammatory response of periodontitis [14], adhere to and invade gingival epithelial cells, interfere with normal physiological metabolism, and inhibit apoptosis [15], which is a potential risk factor for cancer [16]. Gastrointestinal cancer involves common malignant tumors with high morbidity and mortality, low diagnostic rate in the early stage, huge consumption of medical resources [17], high treatment cost for patients at the late stage, and poor effects. Furthermore, the early diagnosis rate of digestive tract tumors is low [18, 19], and advanced treatment costs are high and have poor effects, therefore tumors have become the main healthcare burden of Chinese residents [20]. Domestic and foreign researches have shown a correlation between *P. gingivalis* and digestive tract tumor [21, 22]. This article systematically evaluates the results of recent studies to provide clinical assistance.

### With regard to *P.* gingivalis

*P. gingivalis* is a gram-negative obligate anaerobic bacillus [12], which can express a variety of virulence factors, including trichoderma, gingipains, tetratricopeptide repeat (TPR) sequence protein, extracellular polysaccharides, hemoglobin uptake system, lipopolysaccharides (LPS), etc. [23]. Gingipains and LPS co-activate could affect periodontal tissue immune defense function, and cause inflammation, leading to periodontal tissue destruction and alveolar bone absorption [24].

Gingipains can degrade foreign protein and polypeptide, which provide nutrition to *P. gingivalis* and maintain its growth [12]. *P. gingivalis*can produce virulence factors, such as Outer membrane vesicles (OMVs), E-cadherin, toxin and proteolytic enzyme [25]. *P. gingivalis* OMVs show enriched selectivity in C-terminal domain (CTD)-family proteins, support bacterial cohesion, promote the development of the biological membrane, and function as intermdiates for transporting nonmotile bacteria [26].

As an intracellular pathogenic bacterium, *P. gingivalis* can invade a variety of eukaryotic cells,such as human aortic endothelial cells, human coronary artery endothelial cells (HCAEC) [27], human umbilical vein endothelial cells [28], gingival epithelial cells [15], coronary artery smooth muscle cells (CASMC) [29], epithelial buccal KB cells [30]. After invasion, *P. gingivalis* changes its expressionto avoid the immune defenses of the host and then serves as a reservoir for future reinfection [26, 28]. In addition, it can interact with the host and colonize periodontal tissue [26].

On the basis of different antibody levels of *P. gingivalis* IgG in vivo, periodontitis is divided into none or light(<69EU(Enzyme-linked immunosorbent assay unit)),medium (69.1–119.0 EU) and severe (> 119.0 EU) [31, 32]. After adjusting for risk factors such as age and gender, etc., Ahn J et al [33] discovered that patients with periodontitis exhibit increased cancer mortality (RR = 2.42,95% CI =1.48–3.95). Moreover,the risk still increases even though risk factors, such as smoking, education, race/ethnicity and body mass index are further controlled (RR = 2.28,95% CI = 1.17–4.45), the risk increases with the severity of periodontitis. The mortality rate of patients with periodontitis generally increases with the increase in *P.gingivalis* IgG level [33]. However, the study in Taiwan [34] found that after adjusting the known risk factors, patients with severe periodontitis do not show an increased overall risk of gastrointestinal tumor (HR: 0.99, 95% CI: 0.84–1.16) nor the risk of a single tumor such as esophageal cancer, gastric cancer, small intestinal tumor, colorectal cancer and pancreatic cancer. The reasons for the different findings may be related to differences in disease severity, race and sample size, and differences in risk factors between the two studies.

### *P. gingivalis* and oral squamous cell carcinoma (OSCC)

Oral cancer is the sixth most common cancer in the world and one of the most common cancers in developing countries [35]. OSCC is the most common type of oral cancer with high incidence and recurrence rates and poor prognosis [36, 37]. Patients often experience logopathy and swallowing dysfunction, which seriously affect facial appearance and social interactions [38]. The specific risk factors of OSCC include dietary habits, poor living habits, such as smoking, alcohol abuse, betel nut chewing, DNA carcinogenic virus and genetic factors [39–41],; however, OSCC in approximately 15% of patients still cannot be explained by these factors [42].

Studies have shown that oral flora plays a role in oral cancer [43]. Oral bacterial infection can cause chronic periodontal inflammation, and serum *P.gingivalis* IgG levels are significantly increased in patients with periodontitis compared with normal subjects [31–33]. Pathogenic bacteria such as *P. gingivalis* have been detected in OSCC [44], indicating that periodontitis is probably related to OSCC. After long-term and repeated exposure to *P. gingivalis*, the invasiveness of OSCC cells increases either by IL-8 and MMPs upregulation of [45] or by obtaining stem cell characteristics [46].

After continuous exposure to *P. gingivalis*, human immortalized oral epithelial cells show improved proliferation invasiveness and tumorigenicity improve [47]. The possibility of carcinogenesis increases after the primary cell of a human being is infected by *P. gingivalis* [48]. In addition, continuous infection with *P. gingivalis* causes paclitaxel resistance in OSCC cells, and improved metastatic ability [49]. The relationship of OSCC with *P. gingivalis*, as a periodontitis -dominant bacterium, is a research hotspot that has been extensively reviewed by many researchers [42, 50–52]. Thus, it is a topic that will not be repeated in this paper.

### *P. gingivalis* and esophageal cancer (EC)

EC includes two main types: esophageal adenocarcinoma (ECA) and esophageal squamous cell carcinoma (ESCC), which is dominated by ECA in Western countries, while ESCC is predominant in Eastern countries [53]. EC is the third most common cancer in China, with the fourth highest mortality rate [54]. EC may be related to oral microecology, and Chinese population-based study [55] has shown that poor oral hygiene is closely related to the development of ESCC. A retrospective study in Taiwan [56] revealed that the risk of obtaining EC decreases after the prevention of periodontitis in men (HR = 0.54,95% CI = 0.44–0.66), but no significant statistical difference has been observed in women. Peters B A et al [57] further revealed the increasing *P. gingivalis* can increase the risk of getting ESCC.

Chinese scholars Gao et al [58] discovered that the positive rates of *P. gingivalis* in ESCC, adjacent tissues and normal esophageal tissues were 61, 12% and 0, respectively. *P. gingivalis* infection is predicted to be the dominant factor in esophageal epithelial carcinogenesis and is positively correlated with clinical pathological features such as ESCC tissue differentiation status, metastasis and overall survival [58]. The content of *P. gingivalis* IgG and IgAin 96 ESCC, 50 (esophagitis, EI) and 80 healthy controls serum samples were measured [59]. The sensitivity/specificity of *P. gingivalis* IgG, IgA, and IgG+ IgA for the diagnosis of ESCC is 29.17%/96.90, 52.10%/70.81, and 68.75%/68.46%, respectively. *P. gingivalis* IgA exhibits better diagnostic performance for early ESCC than IgG (54.54% vs. 20.45%). In addition, ESCC patients with high levels of *P. gingivalis* IgG or IgA have poor prognosis, especially those with stage 0-II or lymph node metastasis; furthermore, patients have positive *P. gingivalis* IgG+ IgA present poor prognosis [59]. Thus, *P. gingivalis* may be used for ESCC screening and prognosis monitoring, and is conducive to the early detection of ESCC [58, 59].

### *P. gingivalis* and gastric cancer (GC)

GC is the fourth most prevalent malignant tumor worldwide, and its pathogenesis is a multi-factor, multi-stage process [60]. Studies on the relationship between GC and precancerous lesions have produced inconsistent findings. A meta-analysis [61] showed that tooth loss may be a risk factor for GC. Periodontal pathogen colonization is associated with increased risk of precancerous lesions of gastric cancer [62], thereby suggesting that periodontal pathogens may be associated with GC.

Chronic atrophic gastritis (CAG), intestinal metaplasia (IM) and dysplasia belong to PLGC [63, 64]. In the cross-sectional study by Salazar C R et al [62] on 37 patients with dysplasia, IM or dysplasia and 82 controls. The results showed that the colonization of *P. gingivalis*, *A. actinomycetemcomitans* and *T. denticola* in plaque increased, but it was inconsistent with the DNA level of PLGC. After adjusting for risk factors such as age and gender, Ahn JY et al [33] reported no increase in *P. gingivalis* IgG level in GC (RR = 1.02,95% CI: 0.40–2.55), and this result is similar to the findings when other factors, such as smoking and education, are controlled (RR = 0.99; 95% CI: 0.38–2.58). Yuan et al [22] discovered that *P. gingivalis* cannot survive in environments with high acidity and decreases with increasing acid levels and bacterial flora formation, thereby causing low infection of *P. gingivalis* in the stomach and cardia. The relationship between *P. gingivalis* and GC requires further study.

### *P. gingivalis* and hepatocellular carcinoma (HCC)

HCC is one of the most common malignancies [65] and is the third leading cause of cancer death worldwide [66]. In the vast majority of HCC, viruses and toxic metabolites are observed to cause chronic damage and inflammation, leading to liver fibrosis/cirrhosis [65]. Up till now,risk factors for about 25% HCC are still unknown [67],and bacterial infections may play a role in the development of HCC [66].

Japanese researchers [68] discovered thatpatients with HCC and merged periodontitis have higher Japan integrated stage (JIS) scores and reactive oxygen species (ROS) levels than HCC patients without periodontitis. Han et al [69] reported that periodontitis may participate in the progression of liver diseases, such as nonalcoholic fatty liver disease,cirrhosis, HCC and liver transplantation, but the correlation between periodontitis and liver diseases remains unclear. A prospective cohort study in Finland [70] found that tooth loss among male smokers was correlated with HCC morbidity risk. Compared with patients who have lost 0–10 teeth, those who have lost 11–31 permanent tooth have significantly increased HCC risk (HR 1.42, 95% CI 1.01–1.98). The risk was higher for patients who lost more teeth (HR 1.45, 95% CI 1.00–2.10).

*P. gingivalis* is a pathogenic bacterium that possibly participates in liver diseases formation. In 2009, Nishihara R et al [71] discovered that after KKAy diabetes mice are inoculated with *P. gingivalis*, their blood glucose, serum tumor necrosis factor alpha (TNF-α), and interleukin − 6 (IL-6) are significantly increased,whereas adiponectin is significantly reduced to 35.7%. These findings are similar to results on liver tissues [71]. After treatment with anti-TNF-α antibody, the reaction of KKAy mice to *P. gingivalis* improves, and the lesion size after inoculation is reduced [72]. Moreover, Takano M et al [73] stimulated mouse hepatoma cell Hepa-1.6 with *P. gingivalis* solution and increased the cell TNF-α and IL-6 level; the cell level is significantly decreased after treatment with anti-TNF-α antibody.

Nagao Y et al [74] discovered a positive fimbrillin (fimA) genotype of *P. gingivalis* in patients with hepatitis C (HC) and proposed that periodontitis may be related to the progression of HC; furthermore, controlling oral diseases may be helpful incontrolling hepatic fibrosis. In addition, *gingivalis*lipid Aexhibits anti-MH134 liver cancer activity in C3H/HEN mice [75].*P. gingivalis* possibly participates in hepatic pathological changes through TNF-α and IL-6. However, whether HCC exerts a promoting or restraining effect requires further study.

### *P. gingivalis* and colorectal cancer (CRC)

The relationship between periodontitis and CRC remains unclear. The study of Michaud DS et al [76] on medical staff showed that the tumor morbidity of male patients with periodontitis is increased(+ 13%), whereas patients with severe periodontitis (remaining teeth < 17) exhibit high morbidity rates(+ 45%). Non-smoking male patients with periodontitis have an increased risk of esophageal and head and neck cancer, whereas CRC displays no change [76]. However, Ahn J et al [33] reported that periodontitis is related to the increased in risk of death in patients with CRC. After age and gender risk factors are adjusted, the risk of death from CRC increases (RR = 4.34; 95% CI:1.31–14.44) and remains high after adjusting for smoking, education, race /ethnicity and BMI. (RR = 3.58; 95% CI:1.15–11.16).

Purcell RV and his colleagues [77], for the first time, connected a single bacterium with tumour consensus molecular subtypes (CMS). Their results demonstrated that *P. gingivalis* and *Trichoderma horseshoe* are correlated with CMS_1_, and *F.nucleatum*, *Parvimonasmicra* and *Peptostreptococcusstomatis* are abundant in CMS_1_. Therefore, *P. gingivalis* is candidate bacterium of CRC, but further study is required to verify the correlation.

### *P. gingivalis* and pancreatic cancer (PC)

PC risk factors include smoking, obesity and type 2 diabetes; however, these factors are insufficient to explain the 50% cause of morbidity [10]. A study found that patients with PC often have unbalanced intestinal microbiota [78]. Compared with healthy flora, changes occur in the oral cavity and intestinal and pancreatic tissues of patients with PC [79],indicating a relationship between microecology and PC [80]. Moreover, inflammation may play a role in the development of PC, but the specific mechanism is still unclear [10].

Changes in oral bacterial flora are probably related to the increase in PC morbidity. People with poor oral health have an increased risk of PC disease [81–83], and new research suggests that pathogens may play a role in PC [84]. If this assumption is established, the real cause of pancreatic cancer may be unveiled [85]. The authors [85, 86] speculate that peptide-based arginine deaminase (PAD) enzymes secreted by oral bacterial flora including *P. gingivalis*, *Forsythia bacillus* and *Treponema pallidum* probably cause the gene mutation of P53 and K-ras, which in turn trigger PC. After adjusting for age and gender risk, patients with periodontal diseases present an increased risk of death from PC (RR = 4.99; 95% CI:1.11–22.5), and the risk remains elevated after further adjustment for factors such as smoking, education, race/ethnicity, and BMI (RR = 4.56; 95% CI:0.93–22.29, 34].

Torreshas et al [87] detected the levels of salivary bacteria of patients with PC, identifying 12 eumycota and 139 genus, in which five eumycot as *Proteobacteria, Actinobacteria, Bacteroidetes, Firmicutes* and *Fusobacteria*accounted for 99.3% of the oral bacteria. The proportion of *Leptotrichia* and *Porphyromonas* in the saliva of patients with PC was higher than that in the saliva of healthy controls (*P* < 0.001).

Epidemiological data suggests that *P. gingivalis* may play a role in the pathogenesis of PC [10]. The Fan et al [88] prospective nested case-control study showed that the abundance of. P*. gingivalis* and *Actinobacillus* is increased, whereas that of *Fusobacterium* and *Leptotrichia is* reduced due to the increased PC risk. Michaud et al [21] prospectively investigated the correlation between periodontal antibodies to pathogen and PC risks, and their results revealed that high levels (> 200 ng/ml) of *P. gingivalis* antibody are associated with a twofold increase in PC risk compared with low levels(≤200 ng/ml);furthermore the risk of PC is doubled (OR = 2.14,95% CI, 1.05–4.36), thereby suggesting that periodontitis may increase the risk of PC.

### *P. gingivalis* and other gastrointestinal cancers (gallbladder cancer, cholangiocarcinoma, small intestinal tumors, etc.)

No studies related to periodontitis and cancer have been found in the previous literature. Chou et al [34] first reported that, the risk of cancer for patients with medium periodontitis does not increase compared with that for patients with slight periodontitis (HR = 0.74,95% CI: 0.23–2.34). However, his study was limited by the small sample size. The overall survival rate of Extrahepatic cholangiocarcinoma was greatly lower than those of other cancers [89].Meta-analysis [90] showed that the risk of gallbladder cancer was associated with *Salmonella typhi*, and no related reports on *P. gingivalis* and extrahepatic cholangiocarcinoma were found. Whether *P. gingivalis* is related to the above tumors requires further study.

## Conclusion and future studies

**Fig. 1:**
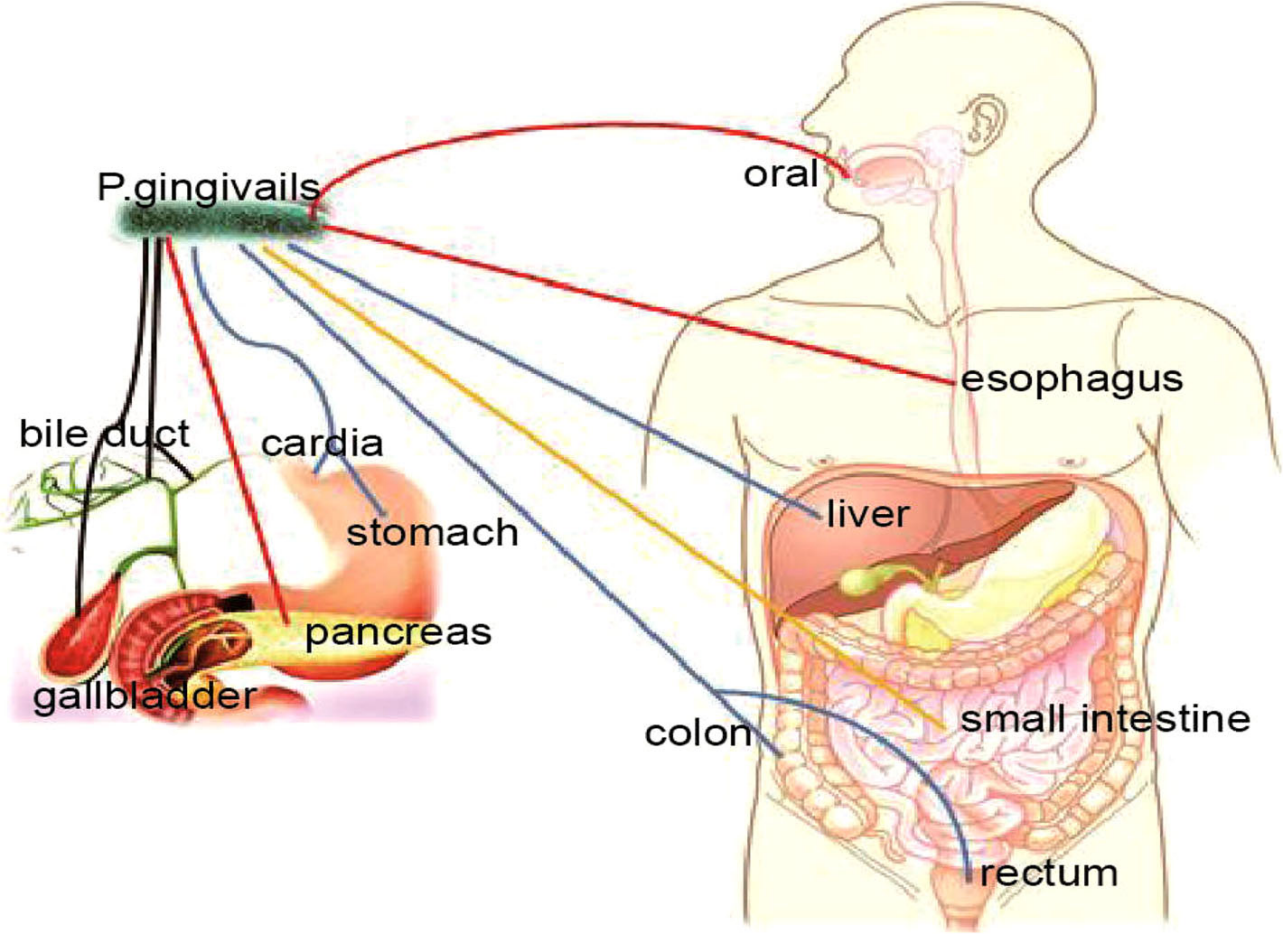
Relationship between *P.gingivalis* and gastrointestinal cancers. Studies have confirmed that *P. gingivalis* could promotetumor progression (red lines). Studies have shown that *P.gingivalis* may promote tumor progression (blue lines). *P.gingivalis* may be associated with tumors (yellow lines). Whether *P. gingivalis* is related to the tumor is currently unclear (black lines). (Some of the color images in Fig. 1 come from the network, and the purpose is only to explain the relationship between *P.gingivalis* and gastrointestinal cancers)

Severe periodontitis, is an important risk factor of gastrointestinal cancer that severely threatens human health. Controlling periodontitis contributes to early cancer prevention. *P. gingivalis* is probably an important risk factor of gastrointestinal cancer, especially for oral cancer, esophageal cancer, colorectal cancer and pancreatic cancer (Fig. 1). Investigations on the relationship among *P. gingivalis*, periodontitis, and gastrointestinal cancer are crucial, but multicenter, prospective, and large-sample studies are necessary.

To date, many studies have supported that periodontitis is a potential risk factor of human diseases. The morbidity rates of periodontal infection and systemic disease have remarkably increasedin previous decades, and the correlation between the diseases and pathogenesis remains unclear [91]. Recently, scientist [92] showed that microorganisms can be used for cancer treatment, but whether *P. gingivalis* is beneficial to human health remains unclear. The pathogenic mechanism of *P. gingivalis* must be further explored to provide a new direction for cancer prevention and treatment, and to improve the health level and quality of life of patients suffering from this disease.

## Data Availability

Not applicable.

## References

[1] Lauritano D, Sbordone L, Nardone M, Iapichino A, Scapoli L, Carinci F (2017). Focus on periodontal disease and colorectal carcinoma. Oral Implantol (Rome).

[2] Moss SF, Blaser MJ (2005). Mechanisms of disease: inflammation and the origins of cancer. Nat Clin Pract Oncol.

[3] Shin HR, Shin A, Woo H, Fox K, Walsh N, Lo YR, Wiesen E, Varghese C (2016). Prevention of infection-related cancers in the WHO Western Pacific region. Jpn J Clin Oncol.

[4] Kaczmarczyk MM, Miller MJ, Freund GG (2012). The health benefits of dietary fiber: beyond the usual suspects of type 2 diabetes mellitus, cardiovascular disease and colon cancer. METABOLISM.

[5] Schwabe RF, Jobin C (2013). The microbiome and cancer. Nat Rev Cancer.

[6] Ahn J, Chen CY, Hayes RB (2012). Oral microbiome and oral and gastrointestinal cancer risk. Cancer Causes Control.

[7] Bodet C, Chandad F, Grenier D (2007). Pathogenic potential of Porphyromonas gingivalis, Treponema denticola and Tannerella forsythia, the red bacterial complex associated with periodontitis. Pathol Biol (Paris).

[8] de Araujo NM, Malo P (2017). Prevalence of periodontitis, dental caries, and peri-implant pathology and their relation with systemic status and smoking habits: results of an open-cohort study with 22009 patients in a private rehabilitation center. J Dent.

[9] Caton JG, Armitage G, Berglundh T (2018). A new classification scheme for periodontal and peri-implant diseases and conditions - introduction and key changes from the 1999 classification. J Periodontol.

[10] Michaud DS (2013). Role of bacterial infections in pancreatic cancer. CARCINOGENESIS.

[11] Mai X, LaMonte MJ, Hovey KM, Freudenheim JL, Andrews CA, Genco RJ, Wactawski-Wende J (2016). Periodontal disease severity and cancer risk in postmenopausal women: the Buffalo OsteoPerio study. Cancer Causes Control.

[12] Plaza K, Kalinska M, Bochenska O, Meyer-Hoffert U, Wu Z, Fischer J, Falkowski K, Sasiadek L, Bielecka E, Potempa B (2016). Gingipains of Porphyromonas gingivalis affect the stability and function of serine protease inhibitor of Kazal-type 6 (SPINK6), a tissue inhibitor of human Kallikreins. J Biol Chem.

[13] Shi J, Leng W, Zhao L, Deng C, Xu C, Wang J, Wang Y, Peng X (2018). Tooth loss and cancer risk: a dose-response meta analysis of prospective cohort studies. Oncotarget.

[14] Rafiei M, Kiani F, Sayehmiri F, Sayehmiri K, Sheikhi A, Zamanian AM (2017). Study of Porphyromonas gingivalis in periodontal diseases: a systematic review and meta-analysis. Med J Islam Repub Iran.

[15] Weinberg A, Belton CM, Park Y, Lamont RJ (1997). Role of fimbriae in Porphyromonas gingivalis invasion of gingival epithelial cells. Infect Immun.

[16] Sayehmiri F, Sayehmiri K, Asadollahi K, Soroush S, Bogdanovic L, Jalilian FA, Emaneini M, Taherikalani M (2015). The prevalence rate of Porphyromonas gingivalis and its association with cancer: a systematic review and meta-analysis. Int J Immunopathol Pharmacol.

[17] Chan J, Chao A, Cheung V, Wong S, Tang W, Wu J, Chan H, Chan F, Sung J, Ng SC. Gastrointestinal disease burden and mortality: A public hospital-based study from 2005 to 2014. J Gastroenterol Hepatol:2018. 10.1111/jgh.14377 29995979

[18] Ghimire B, Singh YP, Timalsina S (2014). Post operative diagnosis of early gastric cancer in a low risk population and the possibility of risk stratified screening. Kathmandu Univ Med J (KUMJ).

[19] Sanchez-Espiridion B, Liang D, Ajani JA, Liang S, Ye Y, Hildebrandt MA, Gu J, Wu X (2015). Identification of serum markers of esophageal adenocarcinoma by Global and targeted metabolic profiling. Clin Gastroenterol Hepatol.

[20] Shao DT, Wei WQ. Progress in research of human microbiota for upper gastrointestinal tumors and precancerous lesions, Chinese J Epidemiol (2018) 382–386. (Chinese). 10.3760/cma.j.issn.0254-6450.2018.03.025 29609258

[21] Michaud DS, Izard J, Wilhelm-Benartzi CS, You DH, Grote VA, Tjonneland A, Dahm CC, Overvad K, Jenab M, Fedirko V (2013). Plasma antibodies to oral bacteria and risk of pancreatic cancer in a large European prospective cohort study. GUT.

[22] Yuan X, Liu Y, Kong J, Gu B, Qi Y, Wang X, Sun M, Chen P, Sun W, Wang H (2017). Different frequencies of Porphyromonas gingivalis infection in cancers of the upper digestive tract. Cancer Lett.

[23] Miller DP, Hutcherson JA, Wang Y, Nowakowska ZM, Potempa J, Yoder-Himes DR, Scott DA, Whiteley M, Lamont RJ (2017). Genes contributing to Porphyromonas gingivalis fitness in abscess and epithelial cell colonization environments. Front Cell Infect Microbiol.

[24] Hajishengallis G (2015). Periodontitis: from microbial immune subversion to systemic inflammation. Nat Rev Immunol.

[25] Xie H (2015). Biogenesis and function of Porphyromonas gingivalis outer membrane vesicles. Future Microbiol.

[26] Gui MJ, Dashper SG, Slakeski N, Chen YY, Reynolds EC (2016). Spheres of influence: Porphyromonas gingivalis outer membrane vesicles. Mol Oral Microbiol.

[27] Rodrigues PH, Belanger M, Dunn WJ, Progulske-Fox A (2008). Porphyromonas gingivalis and the autophagic pathway: an innate immune interaction?. Front Biosci.

[28] Wunsch CM, Lewis JP (2015). Porphyromonas gingivalis as a model organism for assessing interaction of anaerobic Bacteria with host cells. J Vis Exp.

[29] Dorn BR, Harris LJ, Wujick CT, Vertucci FJ, Progulske-Fox A (2002). Invasion of vascular cells in vitro by Porphyromonas endodontalis. Int Endod J.

[30] Lohr G, Beikler T, Hensel A (2015). Inhibition of in vitro adhesion and virulence of Porphyromonas gingivalis by aqueous extract and polysaccharides from Rhododendron ferrugineum L. a new way for prophylaxis of periodontitis?. FITOTERAPIA.

[31] Dye BA, Herrera-Abreu M, Lerche-Sehm J, Vlachojannis C, Pikdoken L, Pretzl B, Schwartz A, Papapanou PN (2009). Serum antibodies to periodontal bacteria as diagnostic markers of periodontitis. J Periodontol.

[32] Noble JM, Borrell LN, Papapanou PN, Elkind MS, Scarmeas N, Wright CB (2009). Periodontitis is associated with cognitive impairment among older adults: analysis of NHANES-III. J Neurol Neurosurg Psychiatry.

[33] Ahn J, Segers S, Hayes RB (2012). Periodontal disease, Porphyromonas gingivalis serum antibody levels and orodigestive cancer mortality. Carcinogenesis.

[34] Chou SH, Tung YC, Wu LS, Chang CJ, Kung S, Chu PH (2018). Severity of chronic periodontitis and risk of gastrointestinal cancers: a population-based follow-up study from Taiwan. Medicine (Baltimore).

[35] Mishra GS, Bhatt SH (2017). Novel program of using village health Workers in Early Detection and Awareness of head and neck cancers: audit of a community screening program. Indian J Otolaryngol Head Neck Surg.

[36] Human Microbiome Project Consortium (2012). Structure, function and diversity of the healthy human microbiome. Nature.

[37] Wang HY, Zhang Y, Zhou Y, Lu YY, Wang WF, Xin M, Guo XL (2016). Rosiglitazone elevates sensitization of drug-resistant oral epidermoid carcinoma cells to vincristine by G2/M-phase arrest, independent of PPAR-gamma pathway. Biomed Pharmacother.

[38] Ni S, Zhu Y, Qu D, Wang J, Li D, Zhang B, Xu Z, Liu S (2017). Morbidity and functional outcomes following free Jejunal flap reconstruction for head and neck Cancer. ORL J Otorhinolaryngol Relat Spec.

[39] Ghantous Y, Abu EI (2017). Global incidence and risk factors of oral cancer. Harefuah.

[40] Ghantous Y, Bahouth Z, Abu EI (2018). Clinical and genetic signatures of local recurrence in oral squamous cell carcinoma. Arch Oral Biol.

[41] Subramanian S, Sridharan N, Balasundaram V, Chaudhari S (2018). Efficacy and safety of nimotuzumab in unresectable, recurrent, and/or metastatic squamous cell carcinoma of the head and neck: a hospital-based retrospective evidence. South Asian J Cancer.

[42] Perera M, Al-Hebshi NN, Speicher DJ, Perera I, Johnson NW (2016). Emerging role of bacteria in oral carcinogenesis: a review with special reference to perio-pathogenic bacteria. J Oral Microbiol.

[43] Lim Y, Totsika M, Morrison M, Punyadeera C (2017). Oral microbiome: a new biomarker reservoir for Oral and oropharyngeal cancers. Theranostics.

[44] Katz J, Onate MD, Pauley KM, Bhattacharyya I, Cha S (2011). Presence of Porphyromonas gingivalis in gingival squamous cell carcinoma. Int J Oral Sci.

[45] Ha NH, Park DG, Woo BH, Kim DJ, Choi JI, Park BS, Kim YD, Lee JH, Park HR (2016). Porphyromonas gingivalis increases the invasiveness of oral cancer cells by upregulating IL-8 and MMPs. Cytokine.

[46] Ha NH, Woo BH, Kim DJ, Ha ES, Choi JI, Kim SJ, Park BS, Lee JH, Park HR (2015). Prolonged and repetitive exposure to Porphyromonas gingivalis increases aggressiveness of oral cancer cells by promoting acquisition of cancer stem cell properties. Tumour Biol.

[47] Geng F, Liu J, Guo Y, Li C, Wang H, Wang H, Zhao H, Pan Y (2017). Persistent exposure to Porphyromonas gingivalis promotes proliferative and invasion capabilities, and tumorigenic properties of human immortalized Oral epithelial cells. Front Cell Infect Microbiol.

[48] Lee J, Roberts JS, Atanasova KR, Chowdhury N, Han K, Yilmaz O (2017). Human primary epithelial cells acquire an epithelial-mesenchymal-transition phenotype during long-term infection by the Oral opportunistic pathogen. Porphyromonas gingivalis Front Cell Infect Microbiol.

[49] Woo BH, Kim DJ, Choi JI, Kim SJ, Park BS, Song JM, Lee JH, Park HR (2017). Oral cancer cells sustainedly infected with Porphyromonas gingivalis exhibit resistance to Taxol and have higher metastatic potential. Oncotarget.

[50] Gholizadeh P, Eslami H, Yousefi M, Asgharzadeh M, Aghazadeh M, Kafil HS (2016). Role of oral microbiome on oral cancers, a review. Biomed Pharmacother.

[51] Wu-chao W, Yafei W, Lei Z (2015). Research progress on the relationship between Porphyromonas gingivalis and oral squamous cell carcinoma. Hua Xi Kou Qiang Yi Xue Za Zhi.

[52] Atanasova KR, Yilmaz O (2014). Looking in the Porphyromonas gingivalis cabinet of curiosities: the microbium, the host and cancer association. Mol Oral Microbiol.

[53] Abnet CC, Arnold M, Wei WQ (2018). Epidemiology of esophageal squamous cell carcinoma. Gastroenterology.

[54] Chen W, Zheng R, Baade PD, Zhang S, Zeng H, Bray F, Jemal A, Yu XQ, He J (2016). Cancer statistics in China, 2015. CA Cancer J Clin.

[55] Chen X, Yuan Z, Lu M, Zhang Y, Jin L, Ye W (2017). Poor oral health is associated with an increased risk of esophageal squamous cell carcinoma - a population-based case-control study in China. Int J Cancer.

[56] Lee YL, Hu HY, Yang NP, Chou P, Chu D (2014). Dental prophylaxis decreases the risk of esophageal cancer in males; a nationwide population-based study in Taiwan. PLoS One.

[57] Peters BA, Wu J, Pei Z, Yang L, Purdue MP, Freedman ND, Jacobs EJ, Gapstur SM, Hayes RB, Ahn J (2017). Oral microbiome composition reflects prospective risk for esophageal cancers. Cancer Res.

[58] Gao S, Li S, Ma Z, Liang S, Shan T, Zhang M, Zhu X, Zhang P, Liu G, Zhou F (2016). Presence of Porphyromonas gingivalis in esophagus and its association with the clinicopathological characteristics and survival in patients with esophageal cancer. Infect Agent Cancer.

[59] Gao SG, Yang JQ, Ma ZK, Yuan X, Zhao C, Wang GC, Wei H, Feng XS, Qi YJ (2018). Preoperative serum immunoglobulin G and a antibodies to Porphyromonas gingivalis are potential serum biomarkers for the diagnosis and prognosis of esophageal squamous cell carcinoma. BMC Cancer.

[60] Kim GH, Liang PS, Bang SJ, Hwang JH (2016). Screening and surveillance for gastric cancer in the United States: is it needed?. Gastrointest Endosc.

[61] Yin XH, Wang YD, Luo H, Zhao K, Huang GL, Luo SY, Peng JX, Song JK (2016). Association between tooth loss and gastric Cancer: a Meta-analysis of observational studies. PLoS One.

[62] Salazar CR, Sun J, Li Y, Francois F, Corby P, Perez-Perez G, Dasanayake A, Pei Z, Chen Y (2013). Association between selected oral pathogens and gastric precancerous lesions. PLoS One.

[63] Sun J, Zhou M, Salazar CR, Hays R, Bedi S, Chen Y, Li Y (2017). Chronic periodontal disease, periodontal pathogen colonization, and increased risk of precancerous gastric lesions. J Periodontol.

[64] Xing J, Min L, Zhu S, Zhang H, Zhao Y, Li H, Zhang Z, Li P, Zhang S (2017). Factors associated with gastric adenocarcinoma and dysplasia in patients with chronic gastritis: a population-based study. Chin J Cancer Res.

[65] Amicone L, Marchetti A (2018). Microenvironment and tumor cells: two targets for new molecular therapies of hepatocellular carcinoma. Transl Gastroenterol Hepatol.

[66] Yu LX, Schwabe RF (2017). The gut microbiome and liver cancer: mechanisms and clinical translation. Nat Rev Gastroenterol Hepatol.

[67] Wang AR, Paletta F, Banki M (2013). A unique presentation of oral metastases from hepatocellular carcinoma. J Oral Maxillofac Surg.

[68] Tamaki N, Takaki A, Tomofuji T, Endo Y, Kasuyama K, Ekuni D, Yasunaka T, Yamamoto K, Morita M (2011). Stage of hepatocellular carcinoma is associated with periodontitis. J Clin Periodontol.

[69] Han P, Sun D, Yang J (2016). Interaction between periodontitis and liver diseases. Biomed Rep.

[70] Yang B, Petrick JL, Abnet CC, Graubard BI, Murphy G, Weinstein SJ, Mannisto S, Albanes D, McGlynn KA (2017). Tooth loss and liver cancer incidence in a Finnish cohort. Cancer Causes Control.

[71] Nishihara R, Sugano N, Takano M, Shimada T, Tanaka H, Oka S, Ito K (2009). The effect of Porphyromonas gingivalis infection on cytokine levels in type 2 diabetic mice. J Periodontal Res.

[72] Takano M, Nishihara R, Sugano N, Matsumoto K, Yamada Y, Takane M, Fujisaki Y, Ito K (2010). The effect of systemic anti-tumor necrosis factor-alpha treatment on Porphyromonas gingivalis infection in type 2 diabetic mice. Arch Oral Biol.

[73] Takano M, Sugano N, Mochizuki S, Koshi RN, Narukawa TS, Sawamoto Y, Ito K (2012). Hepatocytes produce tumor necrosis factor-alpha and interleukin-6 in response to Porphyromonas gingivalis. J Periodontal Res.

[74] Nagao Y, Kawahigashi Y, Sata M (2014). Association of Periodontal Diseases and Liver Fibrosis in patients with HCV and/or HBV infection. Hepat Mon.

[75] Ogawa T, Nakazawa M, Masui K (1996). Immunopharmacological activities of the nontoxic monophosphoryl lipid a of Porphyromonas gingivalis. VACCINE.

[76] Michaud DS, Kelsey KT, Papathanasiou E, Genco CA, Giovannucci E (2016). Periodontal disease and risk of all cancers among male never smokers: an updated analysis of the health professionals follow-up study. Ann Oncol.

[77] Purcell RV, Visnovska M, Biggs PJ, Schmeier S, Frizelle FA (2017). Distinct gut microbiome patterns associate with consensus molecular subtypes of colorectal cancer. Sci Rep.

[78] Signoretti M, Roggiolani R, Stornello C, Delle FG, Capurso G (2017). Gut microbiota and pancreatic diseases. Minerva Gastroenterol Dietol.

[79] Ertz-Archambault N, Keim P, Von Hoff D (2017). Microbiome and pancreatic cancer: a comprehensive topic review of literature. World J Gastroenterol.

[80] Memba R, Duggan SN, Ni CH, Griffin OM, Bashir Y, O'Connor DB, Murphy A, McMahon J, Volcov Y, Ryan BM (2017). The potential role of gut microbiota in pancreatic disease: a systematic review. Pancreatology.

[81] Fitzpatrick SG, Katz J (2010). The association between periodontal disease and cancer: a review of the literature. J Dent.

[82] Meyer MS, Joshipura K, Giovannucci E, Michaud DS (2008). A review of the relationship between tooth loss, periodontal disease, and cancer. Cancer Causes Control.

[83] Michaud DS, Fu Z, Shi J, Chung M (2017). Periodontal disease, tooth loss, and Cancer risk. Epidemiol Rev.

[84] Jacob JA (2016). Study links periodontal disease Bacteria to pancreatic Cancer risk. JAMA.

[85] Ogrendik M (2017). Periodontal pathogens in the etiology of pancreatic Cancer. Gastrointest Tumors.

[86] Ogrendik M (2015). Oral bacteria in pancreatic cancer: mutagenesis of the p53 tumour suppressor gene. Int J Clin Exp Pathol.

[87] Torres PJ, Fletcher EM, Gibbons SM, Bouvet M, Doran KS, Kelley ST (2015). Characterization of the salivary microbiome in patients with pancreatic cancer. PEERJ.

[88] Fan X, Alekseyenko AV, Wu J, Peters BA, Jacobs EJ, Gapstur SM, Purdue MP, Abnet CC, Stolzenberg-Solomon R, Miller G (2018). Human oral microbiome and prospective risk for pancreatic cancer: a population-based nested case-control study. GUT.

[89] Lee SR, Kim HO, Shin JH (2018). The strategy of treatment for mid to distal cholangiocarcinoma after surgical resection. Am Surg.

[90] Nagaraja V, Eslick GD (2014). Systematic review with meta-analysis: the relationship between chronic Salmonella typhi carrier status and gall-bladder cancer. Aliment Pharmacol Ther.

[91] Nagpal R, Yamashiro Y, Izumi Y (2015). The two-way Association of Periodontal Infection with systemic disorders: an overview. Mediat Inflamm.

[92] Guglielmi G (2018). How gut microbes are joining the fight against cancer. NATURE.

